# Everyday executive functioning in pediatric obsessive-compulsive disorder: diagnostic specificity, clinical correlations, and outcome

**DOI:** 10.1186/s12888-023-05111-1

**Published:** 2023-08-24

**Authors:** Frida Rydqvist, Eva Hoff, Daiva Daukantaitè, Matti Cervin

**Affiliations:** 1 Department of Psychology, Lund University, Lund, Sweden; 2 Faculty of Medicine, Department of Clinical Sciences Lund, Child and Adolescent Psychiatry, Lund University, Sofiavägen 2D, Lund, SE-22241 Sweden

**Keywords:** OCD, Children, Adolescents, executive functioning, anxiety, Anxiety disorders, Treatment

## Abstract

**Background:**

Obsessive-compulsive disorder (OCD) typically onsets during childhood or adolescence and difficulties with executive functioning (EF) may be involved in its onset and maintenance. Yet, few studies have examined everyday EF difficulties in youth with OCD and no study has compared EF in youth with OCD to EF in youth with anxiety disorders, leaving the diagnostic specificity of EF unclear.

**Methods:**

In this study, parents of treatment-seeking children and adolescents with OCD (*n* = 96, *M*_age_ = 13.3, *SD* = 2.7, 59% girls) or anxiety disorders (*n* = 67, *M*_age_ = 14.0, *SD* = 2.6, 78% girls) reported on their children’s everyday EF using the Behavior Rating Inventory of Executive Function (BRIEF) measure.

**Results:**

Compared to community youth, the two clinical groups showed moderately elevated EF deficits but did not differ significantly from each other. EF deficits were not associated with the major symptom dimensions of OCD, age of OCD symptom onset, duration of OCD symptoms, and OCD severity, and did not predict treatment outcome in OCD.

**Conclusions:**

Compared to peers, youth with OCD show moderate difficulties with EF, but very similar difficulties are seen in youth with anxiety disorders, and it is unclear whether these difficulties are of clinical relevance. Among youth with OCD, EF difficulties were not differentially associated with the major symptom dimensions of OCD, which is inconsistent with findings from adults. Difficulties with EF did not predict treatment outcome, indicating that integrating EF modules into OCD treatment may be of limited value, although EF may be important for treatment planning in individual cases.

**Supplementary Information:**

The online version contains supplementary material available at 10.1186/s12888-023-05111-1.

## Background

Obsessive-compulsive disorder (OCD) is a mental disorder with an estimated prevalence of 1–3% in the general population [1, 2]. OCD is characterized by distressing and intrusive thoughts, urges or images (obsessions), resulting in compulsive acts carried out to reduce the discomfort, distress or anxiety caused by obsessions [3–5]. More than half of all individuals with OCD experience their first symptoms before adulthood [6], making research on pediatric OCD important. Pediatric OCD is known for its heterogeneity, but symptoms can be divided into thematically coherent symptom dimensions, with the most replicated dimensions being disturbing thoughts/checking, contamination/cleaning, and symmetry/ordering [7–9]. Co-occurring mental disorders are common in pediatric OCD and often include anxiety disorders, depressive disorders, and neurodevelopmental disorders such as attention deficit hyperactivity disorder (ADHD) and autism spectrum disorder [2].

It has been proposed that behavioral and executive dysregulation may be core deficits underlying OCD, and that cognitive control may be a key endophenotype in OCD [10]. Cognitive flexibility, as well as cognitive and behavioral control and regulation, all belong under the umbrella term known as executive functions (EF), which are a set of self-regulatory, higher cognitive functions responsible for emotional and behavioral regulation and the ability to execute goal-directed actions related to every-day functioning and long-term goals [11]. EF is described as a multidimensional construct and includes several interconnected, yet distinguishable neurocognitive processes [12, 13] closely intertwined with the ability to exhibit self-control in areas such as organization, planning, affect regulation, initiation and overall attention [11].

EF develops and matures over time, from early childhood into early adulthood [14], suggesting that younger children tend to experience more EF-related difficulties compared to adolescents and adults [15]. Given the early onset of OCD, research on EF in pediatric OCD may provide important insight into processes and mechanisms involved in the onset and maintenance of the disorder.

Previous research on pediatric OCD and EF is limited and with contradictory results, often contrasting results found in adults with OCD. A systematic review and meta-analysis synthesized 11 studies on EF in pediatric OCD [16]. Results were categorized into nine EF subdomains: planning, response inhibition/interference control, set shifting/cognitive flexibility, verbal memory, nonverbal memory, processing speed, working memory, visuospatial functions, and attention. All included studies measured EF through performance-based neuropsychological tasks and did not include rating scales of everyday EF skills. Small degrees of underperformance on most subdomains were identified, except for the response inhibition and interference control subdomains, where performances were similar in the OCD group and comparison groups from the general population. While there were some indications of a small to moderate degree of underperformance in planning in youth with OCD, no meta-analytic comparison was statistically significant, leading to the conclusion that task-based neuropsychological deficits in EF seem to have no clear association with pediatric OCD. However, the study also acknowledged that few studies were available and that more research is needed to consolidate the understanding of neuropsychological functioning and EF in youth with OCD [16]. A recent study, also using performance-based EF tasks, investigated several neurocognitive domains in youth with OCD, their unaffected siblings, and parents [17]. Results showed that cognitive flexibility and inhibitory control may be two candidate endophenotypes in pediatric OCD, while no significant familial effects were found for the other EF subdomains.

Consequently, research on EF in pediatric OCD shows inconsistent results. A potential explanation is the lack of a consensus regarding how to define and measure EF, which is a multidimensional construct [18]. EF is typically measured through performance-based tasks [11], a method that is not always optimal. For example, patients with frontal lobe damage and clear daily life impairments have managed to perform normally or above normal on traditional neuropsychological tasks of language, memory, perception, and EF [19]. Thus, real-world, observational tasks have been suggested to be a more effective and ecologically valid method to capture EF impairments than the sole use of performance-based EF tasks conducted in lab-settings [20]. Generally, performance-based EF tasks do not seem to capture the same constructs as rating scales or direct observations of EF in daily life [20]. This is important since distinct EF processes (e.g., response inhibition) have been suggested as candidate endophenotypes in OCD [10]. Thus, conflicting results regarding EF and pediatric OCD may be explained by the extensive reliance on performance-based EF measures.

Further, no previous studies have compared EF in pediatric OCD to other EF in other mental disorders, leaving it uncertain whether deficits in EF are linked to OCD specifically or are transdiagnostic in nature (i.e., related to many different forms of symptoms and disorders). Moreover, associations between EF and the known symptom heterogeneity of pediatric OCD remain unclear but could help explain inconsistent and conflicting results in previous research. Differences in EF across different OCD symptom dimensions have been observed in adults [21], but few studies have examined EF across OCD symptom dimensions in pediatric OCD. However, a recent study that included parent ratings of EF using the Behavior Rating Inventory of Executive Function (BRIEF) [22] in combination with EF tasks, showed no support for the relevance of EF in relation to the symptom heterogeneity of pediatric OCD [23]. Last, few studies have examined whether EF predicts treatment outcome for youth with OCD, with current studies yielding inconsistent results [24–26]. The association between EF and treatment outcome is important as such an association could imply that integrating EF modules into OCD treatment may improve outcomes.

Regarding diagnostic specificity of EF in relation to pediatric OCD, a comparison to pediatric anxiety disorders is of relevance. OCD was long considered an anxiety disorder but was included in its own chapter in DSM-5, where it was acknowledged that OCD shares features with anxiety disorders (fear, anxiety, and avoidance), but that there are also elements that make OCD distinct from anxiety disorders (e.g., compulsivity). Comparing EF in youth with OCD to EF in youth with anxiety disorders can help improve the understanding of the role of EF in OCD and whether some EF features are specific to OCD. Research on EF in youth with anxiety disorders is sparse compared to research on EF in pediatric OCD. The available studies suggest that pediatric anxiety disorders may be associated with some EF deficits, particularly inhibition difficulties, although findings are mixed [27].

As mentioned, research on rating-based EF in youth with OCD is limited, but the few published studies have shown worse EF among youth with OCD compared to healthy controls [23, 26]. For example, one study [23] found that all three subgroups of youth with OCD (symmetry/hoarding, harm/sexual, and contamination/cleaning symptoms) had increased parent-reported difficulties with inhibition and shifting compared to healthy controls, while no associations were found between EF scores (ratings or task-based performance) and symptom dimensions. A recent study [28] used both EF tasks and rating scales (BRIEF) to assess EF in OCD and found that youth with OCD demonstrated greater executive dysfunction in real-life contexts (as measured with BRIEF) compared to their EF ability on performance-based tasks in controlled settings. This finding provides further support for the notion that EF difficulties in youth with OCD may be underestimated when relying solely on performance-based EF. Further, youth with OCD had significantly higher EF scores (indicating more difficulties) than healthy controls, with large effect sizes for Shift (Cohen’s *d* = 1.36), Working memory (Cohen’s *d* = 0.92), Planning (Cohen’s *d* = 0.89), and Inhibition (Cohen’s *d* = 0.78). Of note, BRIEF scores were not associated with OCD severity.

The aim of this study is to investigate everyday EF in pediatric OCD. We will analyze parent-ratings of everyday EF using the BRIEF in a sample of children and adolescents with OCD and compare their scores to norm scores from peers and scores from a sample of youth with anxiety disorders but no OCD. We will also examine whether EF deficits are more common in certain OCD symptom dimensions (i.e., disturbing thoughts/checking, contamination/cleaning and symmetry/ordering), with symptom dimensions being assessed using a validated interview. Finally, we will examine whether EF predicts treatment outcome in OCD. Based on previous research [28], we expect that EF deficits are elevated among youth with OCD compared to peers and that differences are largest for the EF domains of Shift, Working memory, Planning, and Inhibition. Based on previous research [23, 28], we do not expect EF to be statistically significantly linked to OCD symptom dimensions. With respect to the comparison to youth with anxiety disorders and treatment outcome for youth with OCD, we proceed without predefined hypotheses based on non-existent studies (comparison to youth with anxiety disorders) and inconsistent results in previous studies (treatment outcome).

## Methods

### Participants

Participants were 163 children and adolescents recruited from a specialized child and adolescent outpatient clinic in southern Sweden where they were part of a larger project examining emotional and cognitive processes in pediatric OCD. Approximately two thirds (67%) were female, and the mean age was 13.6 years (SD = 2.7). Ninety-six participants had OCD as their principal disorder and 67 had an anxiety disorder as their principal disorder (generalized anxiety disorder: 38%, panic disorder: 11%, separation anxiety disorder: 11%, specific phobia: 12%, social anxiety disorder: 28%). None of the participants with an anxiety disorder met diagnostic criteria for OCD. Thirty-four participants (21%) had co-existing neurodevelopmental disorders: autism spectrum disorder (6%) and ADHD (18.0%). Sociodemographic and clinical information for the OCD and anxiety disorder samples are presented in Table 1. All participants and their caregiver/s provided written informed consent/assent, and the study was approved by the regional ethics committee at Lund University, Lund, Sweden (Dnr 2015/663) and all study procedures were performed in accordance with relevant guidelines and regulations.

**Table 1:** Sociodemographic and clinical data across groups

	OCD	Anxiety Disorders	Total
*n*	96	67	163
Girls, *n* (%)	57 (59%)	52 (78%)	109 (67%)
Age, *M* (*SD*)	13.3 (2.7)	14.0 (2.6)	13.6 (2.6)
Any neurodevelopmental disorder, *n* (%)	22 (23%)	12 (18%)	34 (21%)
ADHD, *n* (%)	17 (18%)	11 (17%)	28 (17%)
Autism spectrum disorder, *n* (%)	7 (7%)	2 (3%)	9 (6%)
Ongoing major depression, *n* (%)	10 (11%)	17 (26%)	28 (17%)
CGAS	51.5 (3.1)	54.1 (5.6)	52.4 (4.3)
CY-BOCS, *M* (*SD*)	23.2 (4.2)	-	-
OCD severity according to CY-BOCS at intake			
Mild	34 (37%)	-	-
Moderate	52 (57%)	-	-
Severe	6 (7%)	-	-
CY-BOCS, follow-up, *M* (*SD*) [*n* = 83]	15.7 (6.5)		
Anxiety disorders, *n* (%)	47 (50%)	67 (100%)	114 (70%)
Family economy, good or better, *n* (%)*	72 (80%)	40 (78%)	112 (79%)
Living with both parents, *n* (%)**	66 (69%)	38 (66%)	101 (68%)
Age at OCD symptom onset, *M* (*SD*)	8.02 (2.73)	-	-
Duration of OCD symptoms, *M* (*SD*)	5.23 (3.14)	-	-

*Notes*. * Self-reported by parents to indicate the overall economic situation around the child; missing data for 6 participants in the OCD group and 16 participants in the anxiety disorders group. ** Self-reported by parents or youth indicating the living arrangement of the participant, other options include joint custody, seeing one parent only on weekends, no contact with one of the parents, and other living arrangement (e.g., foster care); missing data for 9 participants in the anxiety disorders group. ADHD = Attention Deficiency Hyperactivity Disorder. CY-BOCS = Children’s Yale-Brown Obsessive Compulsive Scale

### Measures

**MINI-KID.** MINI-KID is a structured diagnostic interview that assesses the most common mental disorders in youth [29]. In the present study, the MINI-KID was used to assess diagnostic status for all participants at intake, including the presence of major depression, and the interview was carried out by clinical psychologists trained in using the instrument.

**Children’s global assessment scale (CGAS).** As part of the clinical interview, each participant was scored using the CGAS. The CGAS is a measure of psychosocial functioning ranging from 1 to 100 that integrates psychological, social, and academic functioning into an overall impairment score which is not restricted to specific symptoms. The measure has shown adequate validity and reliability in youth with mental disorders [30].

**BRIEF.** The BRIEF is a rating scale for the assessment of EF in 5-18-year-old children and adolescents [22]. The BRIEF has three versions: a self-report form, a parent-report form, and a teacher-report form, with eight scales included in each version (Inhibit, Shift, Emotional control, Initiate, Working memory, Plan/Organize, Organization of materials, Monitor), two broader indexes (Behavioral regulation and Metacognition) as well as an overall score, the Global Executive Composite. In this study, the parent version was used. Both raw scores and age and sex-adjusted normative scores transformed to t scores (*M* = 50, *SD* = 10) were analyzed. The BRIEF was completed by parents at intake. The parent-version of the BRIEF has previously been subject for evaluation among clinical youth samples and an exploratory factor analysis has supported an 8-factor model with two second order factors in both typically developing and mixed clinical samples [22].

To the best of our knowledge, the psychometric properties of the BRIEF has not been examined in a Swedish clinical context and we conducted a psychometric evaluation using the present samples (see the Supplementary for a methodological description). In short, the proposed BRIEF factor structure (8 first-order factors and 2 broader factors) had adequate to good model/data fit and much better fit than a unidimensional factor structure. It also had a similar fit to a model where all first-order factors were allowed to correlate freely, see the Supplementary for detailed results. The internal consistency of the items of the 8 first-order factors was good to excellent for all factors: Inhibit (*a* = 0.93), Shift (*a* = 0.85), Emotional control (*a* = 0.94), Initiate (*a* = 0.84), Working memory (*a* = 0.94), Plan/ Organize (*a* = 0.93), Organization of materials (*a* = 0.92), and Monitor (*a* = 0.86).

**Children’s Yale-Brown Obsessive Compulsive Scale (CY-BOCS).** The CY-BOCS is the most common severity measure of OCD in youth [31]. It rates obsessions and compulsions separately according to time, distress, impairment, resistance, and control using 0–4 Likert items (5 items for obsessions and 5 for compulsions). This yields a total score of 0 to 40, with higher scores indicating more severe OCD. The clinical threshold of OCD is 14 points, scores above 21 correspond to moderate OCD, and scores between 30 and 40 indicate severe OCD [32]. For participants with OCD, trained interviewers conducted CY-BOCS at intake and at follow-up. In all cases, children/adolescents were present during the interview and in most cases, one or both parents were also present.

**Dimensional Yale-Brown Obsessive Compulsive Scale (DY-BOCS).** The DY-BOCS is an interview-based measure that assesses OCD symptom severity across the major symptom dimensions of OCD [33, 34]. In the present study, severity scores for disturbing thoughts/checking, symmetry/ordering, and contamination/cleaning were used. In DY-BOCS, the interviewer scores the severity within each symptom dimension using three 0–5 items capturing time, interference and distress. This generates a total score of 0–15 for each symptom dimension with higher scores indicating more severe symptoms within that dimension. Symptoms within each dimension were assessed at intake using a semi-structured interview that has showed validity and utility in Swedish youth with OCD [33]. During the DY-BOCS interview, age at OCD symptom onset was also assessed.

### Procedure

The diagnostic status of participants was examined at intake using the MINI-KID [29]. All participants with OCD were assessed with the CY-BOCS at intake and at follow-up (*n* = 83, 90% of OCD participants). The average follow-up time for OCD participants was 13.31 months (SD = 6.69). All OCD participants had been offered exposure-based cognitive behavioral therapy (CBT), 78% had engaged in CBT, and 31% had been treated with a combination of CBT and selective serotonin reuptake inhibitors. The mean number of CBT sessions was 9.3 (SD = 6.1).

### Statistical analysis

To examine whether the OCD and anxiety disorder groups differed from peers, we used one-sample t-tests in which we compared the age- and sex-based BRIEF T-scores to a normative T-score of 50, which is the mean of the population. T-scores are a special kind of standardized scores, with a mean of 50 and a standard deviation of 10, which is often used for psychological normative data and result from transforming raw scores to standardized scores. A 95% confidence interval (CI) for the difference was used to examine whether the groups differed statistically significantly from the mean of the general population. Analyses were run with and without participants with neurodevelopmental disorders (ADHD and/or autism spectrum disorder) to examine whether the presence of these disorders could explain possible differences compared to the general population.

To examine whether the OCD and anxiety groups differed on the different EF domains, linear regression analyses accounting for age, sex, and the presence of neurodevelopmental disorders were conducted using each of the EF domains (raw scores) as the dependent variable and group (OCD vs. anxiety disorders), age, sex, and the presence of neurodevelopmental disorders as independent variables. To examine whether EF was associated with the major OCD symptom dimensions (i.e., disturbing thoughts/checking, symmetry/ordering, and contamination/cleaning measured via DY-BOCS), regression analyses were conducted where the DY-BOCS dimensional severity scores were regressed onto the BRIEF factors (raw scores), age and sex. These analyses were only conducted in the OCD group as only these participants had severity scores for the OCD dimensions. Associations between OCD severity at intake, age at OCD symptom onset, and duration of OCD symptoms and EF was examined by correlating these variables with all EF scores. T-scores were used to account for age and sex effects.

To predict treatment outcome, we conducted linear regression with the post-treatment CY-BOCS score as the dependent variable and BRIEF, age, sex, neurodevelopmental status, and the CY-BOCS intake severity as independent variables. To adjust for multiple comparisons, we used an alpha value of 0.01 as an indicator of statistical significance in all models.

## Results

### Differences compared to peers

**Fig. 1:**
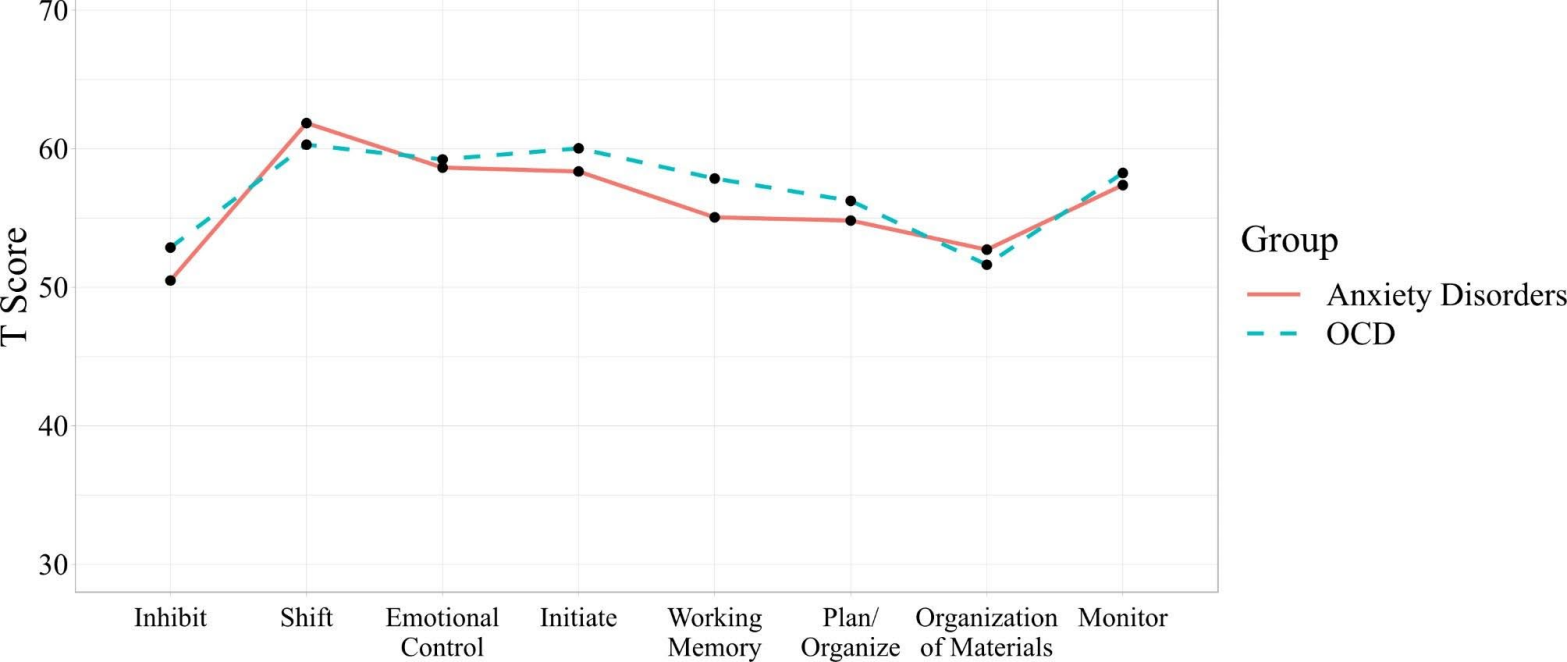
BRIEF profiles for the OCD and anxiety disorder groups; the population mean is 50

Age- and sex-transformed BRIEF T-scores for the OCD and anxiety disorder groups were compared to a general T-score of 50 (the population mean) using one-sample t-tests. Results are presented in Table 2, and in Fig. 1 is an illustration of the BRIEF profiles for youth with OCD and anxiety disorders. Youth with OCD and anxiety disorders differed significantly from the general population on all EF domains except Organization of materials (both groups) and Inhibit (anxiety disorders). In both groups, effect sizes were largest for Shift (*d* = 0.90 and 0.95 for the OCD and anxiety disorder groups, respectively), Emotional control (*d* = 0.77 and 0.68), and Initiate (*d* = 0.82 and 0.70).

**Table 2:** Results from one-sample t-tests for the OCD and anxiety groups EF T-scores compared to a normative score of T = 50

Executive functioning domain	OCD (*n* = 92–96)*, *M* (*SD*)	*p* for Comparison with T = 50	Cohen’s *d*	Cohen’s *d* without neurodevelopmental
Inhibit	52.87 (9.69)	0.005	0.30	0.16
Shift	60.29 (11.47)	< 0.001	0.90	0.79
Emotional Control	59.23 (11.97)	< 0.001	0.77	0.70
Initiate	60.03 (12.23)	< 0.001	0.82	0.72
Working memory	57.85 (12.16)	< 0.001	0.65	0.56
Plan/organize	56.24 (12.10)	< 0.001	0.52	0.36
Organization of materials	51.63 (10.53)	0.134	0.15	0.42
Monitor	58.25 (17.91)	< 0.001	0.46	0.29
Behavior Regulation Index	58.29 (10.31)	< 0.001	0.80	0.70
Metacognition Index	57.21 (11.38)	< 0.001	0.63	0.49
Global Executive Composite Index	58.11 (10.85)	< 0.001	0.75	0.59
	Anxiety Disorder (*n* = 66–67)*, *M* (*SD*)			
Inhibit	50.49 (11.51)	0.727	0.04	-0.15
Shift	61.85 (12.44)	< 0.001	0.95	0.85
Emotional Control	58.64 (12.70)	< 0.001	0.68	0.62
Initiate	58.36 (11.99)	< 0.001	0.70	0.60
Working memory	55.05 (11.40)	< 0.001	0.44	0.31
Plan/organize	54.82 (11.19)	< 0.001	0.43	0.28
Organization of materials	52.72 (11.16)	0.05	0.24	0.16
Monitor	57.38 (15.91)	< 0.001	0.46	0.33
Behavior Regulation Index	57.77 (11.81)	< 0.001	0.66	0.56
Metacognition index	56.00 (11.12)	< 0.001	0.46	0.40
Global Executive Composite Index	57.03 (11.19)	< 0.001	0.66	0.50

*Notes.* * Participants with missing data on more than two items per subscale were excluded

When excluding those with neurodevelopmental disorders, effect sizes were somewhat reduced but largely intact and there was still a statistically significant difference compared to the mean of the general population (except that Inhibition in the OCD group was no longer statistically significantly different from this mean). To examine the possibility that major depression could impact EF scores, we conducted independent samples t-tests comparing those with versus without major depression (full clinical sample) on all EF variables. No significant differences emerged (all *p*s > 0.19). We also examined whether EF was associated with overall functioning (CGAS) and no significant associations were present (r*s* = − 0.15 to − 0.02., all *p*s > 0.07).

### Differences between those with OCD and those with anxiety disorders

Regression models accounting for differences in age, sex, and the presence of neurodevelopmental disorders showed that the OCD and anxiety disorders groups did not differ significantly from each other on any EF domain (raw scores were used and positive *β* values indicate more EF difficulties in the OCD group): inhibit (*β* = 0.05, *p* = .54), shift (*β* = -0.08, *p* = .32), emotional control (*β* = 0.01, *p* = .95), initiate (*β* = 0.02, *p* = .84), working memory (*β* = 0.09, *p* = .25), plan/organize (*β* = 0.03, *p* = .66), organization of materials (*β* = -0.08, *p* = .29), monitor (*β* = -0.02, *p* = .84), behavior regulation index (*β* = -0.01, *p* = .88), metacognitive index (*β* = 0.02, *p* = .81), global executive composite index (*β* = 0.01, *p* = .95).

Because around half of the participants with OCD also met criteria for an anxiety disorder, we divided the full sample into three groups: [a] OCD and a co-occurring anxiety disorder, [b] an anxiety disorder but no OCD, and [c] OCD but no anxiety disorder. We conducted one-way ANOVAs to compare the three groups. No significant differences on any EF domain emerged (all ps > 0.07).

### Clinical correlates among youth with OCD

The results for associations between EF and the major symptom dimensions of OCD showed no statistically significant associations: disturbing thoughts/checking (all *p*s > 0.22), symmetry/ordering (all *p*s > 0.08), contamination/cleaning (all *p*s > 0.18). None of the EF domains was significantly correlated with OCD severity at intake (CY-BOCS total score; r*s* = − 0.16 to 0.19, all *p*s > 0.07), age of OCD symptom onset (r*s* = − 0.19 to 0.04, all *p*s > 0.07), or duration of OCD symptoms (r*s* = − 0.04 to 0.13, all *p*s > 0.23).

### EF as a predictor of naturalistic treatment outcome

Each EF domain (raw scores) as well as the broad EF indexes were included alongside age, sex, the presence of a neurodevelopmental disorder, and OCD severity at intake as predictors of post-treatment OCD severity (CY-BOCS) in 11 separate models (one for each EF domain/index). None of the EF domains/indexes was a statistically significant predictor of treatment outcome (all *β*s < 0.18; all *p*s > 0.11).

## Discussion

The present study examined everyday EF in youth with OCD. To our knowledge, this is the first study to compare EF difficulties in pediatric OCD to EF difficulties in youth with anxiety disorders. First, compared to norm scores from peers, youth with OCD and anxiety disorders showed significant differences on all domains except Organization of materials (both groups) and Inhibition (youth with anxiety disorders). However, differences were mostly moderate, with the largest effect sizes for both groups emerging for Shift, Emotional control, and Initiate. Only Shift was included in our hypotheses about which EF difficulties would be most elevated in OCD and this finding is in line with a recent study where OCD probands, their unaffected siblings and parents showed deficiencies in cognitive flexibility and inhibitory control [17]. In our study, cognitive flexibility is mirrored by the Shift subscale, which assesses the ability to adjust behavior flexibly to changing demands of a situation [22]. A meta-analysis on cognitive inflexibility in adults with OCD found deficits in cognitive flexibility [35], which is also in line with our findings. The link between cognitive flexibility and OCD is intuitive as OCD is characterized by non-flexible behaviors [3–5]. However, in the present study, youth with anxiety disorders showed similar deficits in cognitive flexibility, indicating that this is not unique to pediatric OCD.

Moderate deficits compared to peers were found for Emotional control and Initiate in both groups. These scales capture abilities related to modulation of emotional responses (Emotional control), the ability to begin a task or activity as well as the capacity to independently generate ideas or problem-solving strategies (Initiate) [22]. Research on problem-solving strategies in OCD is scarce, however, one study revealed no impaired problem-solving strategies in adults with OCD, measured using performance-based EF tasks [36]. Regarding emotion regulation, our findings are in line with evidence indicating that difficulties with emotion regulation is related to several psychiatric disorders in adults, including OCD, where it is often characterized by diminished reappraisal abilities and increased use of suppression strategies [37]. Our results expand this body of research by showing that overall EF deficits are not specific for OCD but extend to pediatric anxiety disorders.

Of note, the BRIEF profiles for the OCD and anxiety disorder groups were very similar with almost identical mean scores across the different subscales, with most elevated scores on Shift, Emotional Control, and Initiate. When controlling for co-occurring neurodevelopmental disorders (i.e., ADHD and autism spectrum disorder), effect sizes were slightly decreased but differences remained statistically significant compared to peers, except for Inhibition in OCD. These results indicate that even when EF in youth with neurodevelopmental disorders, where EF difficulties are prominent [38], are accounted for, youth with OCD and anxiety disorders still exhibit EF difficulties compared to peers. It is unclear whether these difficulties are directly linked to OCD/anxiety disorders, expressions of subclinical neurodevelopmental traits, or both.

No differences in EF were found when comparing youth with OCD and anxiety disorders. In fact, both groups showed very similar EF profiles. There is some evidence that the major symptom dimensions of OCD are underpinned by partly different neural substrates [39], and hypotheses for EF deficits in OCD largely stem from observed deviations in neural circuits known to be involved in EF [40]. However, in line with a previous study on EF and symptom dimensions in pediatric OCD [23], we found no significant associations between the two. This contrasts findings in adults, where contamination/cleaning symptoms have been associated with better EF [21].

Overall, the findings of the present study do not indicate a strong link between EF and pediatric OCD. First, youth with OCD and anxiety disorders did not differ from each other. Second, differences compared to peers were generally moderate. Third, there was no association with overall OCD severity or the major OCD symptom dimensions. Fourth, EF was not linked to naturalistic treatment outcome. Taken together, these results suggest that EF deficits may be more transdiagnostic than disorder-specific, which is largely in line with research about the role of EF in mental disorders in children and adolescents [41]. That EF did not predict treatment outcome indicates that integrating EF modules into OCD treatment may be of limited value. However, in individual cases, EF deficits may be important for treatment planning. Our results about EF and treatment outcome partially contrast findings from a recent study, where difficulties with emotion regulation were associated with a poorer response to treatment in youth with OCD [42].

Several limitations merit mentioning. First, we only used parent-rated measures of EF. Future research should consider combining different raters and measures (e.g., teachers, self-report, observational measures, and tasks), not the least since ratings of EF in daily life have been shown to differ substantially from EF measured using performance-based tasks [20]. Second, treatment was not delivered under controlled conditions and follow-up assessments were carried out on average more than a year after treatment initiation. Although this makes it hard to draw conclusions about whether EF moderates outcome of highly structured and time-limited OCD interventions, it provides evidence for that EF does not seem to moderate more long-term outcomes of broader naturalistic treatment of youth with OCD. Third, we used normative scores derived from an American youth population. A comparison group of Swedish community children and adolescents or Swedish normative scores would have been preferred but was not available and the resources available to the project did not allow for producing Swedish norm scores. An alternative would have been to recruit a non-clinical comparison group, but such an approach has drawbacks as it is challenging to secure representativity. Fourth, to examine whether neurodevelopmental status explained group differences in EF, we used established neurodevelopmental diagnoses, which do not appreciate the dimensional nature of neurodevelopmental symptoms in youth and the study did not collect dimensional scores on neurodevelopmental traits [43].

## Conclusions

This study showed that youth with OCD have deficits in everyday EF compared to peers and that these differences are not fully explained by the presence of neurodevelopmental disorders. Nevertheless, our results suggest only moderate EF deficits, which are equally apparent in youth with anxiety disorders, not associated with OCD severity or the major symptom dimensions of OCD, and not associated with naturalistic treatment outcome. Taken together, our findings indicate that EF deficits may have little relevance for the clinical management of pediatric OCD, and while prospective studies are needed, it is unclear whether EF can offer unique insights into its etiology.

### Electronic supplementary material

Below is the link to the electronic supplementary material.

Supplementary Material 1: Everyday executive functioning in pediatric obsessive-compulsive disorder

## Data Availability

The datasets used and/or analysed during the current study are available from the corresponding author on reasonable request.
