# Supplementary material for: Everyday executive functioning in pediatric obsessive-compulsive disorder: diagnostic specificity, clinical correlations, and outcome
Source: BMC Psychiatry. 2023 Aug 24;23:622. doi: 10.1186/s12888-023-05111-1 (PMC10464101; doi:10.1186/s12888-023-05111-1)
Supplement: Supplementary file 1 — Supplementary Material 1: Everyday executive functioning in pediatric obsessive-compulsive disorder [file 12888_2023_5111_MOESM1_ESM.docx]

**Supplemental material**

for

Everyday executive functioning in pediatric obsessive-compulsive disorder:

Diagnostic specificity, clinical correlations, and outcome

Page 1. Supplemental methods

Page 2. Supplemental Table 1

**Supplemental methods**

We examined the psychometric properties of BRIEF by running confirmatory factor analysis (CFA) of the proposed BRIEF model with eight first-order factors (Inhibit, Shift, Emotional control, Initiate, Working memory, Plan/Organize, Organization of materials and Monitor) and two second-order factors (Behavioral regulation and Metacognition). The R library *lavaan* was used for these analyses using diagonally weighted least squares estimation which is specifically designed for handling ordinal data. The model/data fit of the proposed eight-factor model was compared to model/data fit of (1) a unidimensional one-factor model in which associations among items are explained by a single broad EF factor and (2) a model where the proposed first-order factors were allowed to correlate freely. The latter model was used to examine the degree to which the second-order factors of Behavioral Regulation and Metacognition were adequate. Model/data fit was evaluated using the following fit indexes: Confirmatory Fit Index (CFI), Root Mean Square Error of Approximation (RMSEA), Standardized Mean Square Residual (SRMR), and Tucker-Lewis fit Index (TLI). Adequate model fit is indicated by a lower chi-square value, higher CFI/TLI (values > 0.90 are indicative of adequate fit), and lower RMSEA and SRMR (values < 0.06 and 0.08, respectively, are indicative of good fit) (1). Scaled fit indexes were computed because of the ordinal items. A global evaluation of the fit indexes was conducted. Internal consistency of each scale was computed within the CFA using ordinal alpha with estimates above .70 indicating adequate internal consistency.

**Supplemental Table 1.**

*Model/data fit for the proposed BRIEF factor structure and a unidimensional structure used as a comparison.*

|  | |  | *χ2* | *df* | *p* | CFI | TLI | RMSEA | SRMR |
| --- | --- | --- | --- | --- | --- | --- | --- | --- | --- |
| **BRIEF models** | | |  |  |  |  |  |  |  |
| 1 | Proposed factor structure with 8 first-order and two second-order factors | | 3238 | 2475 | < .001 | .925 | .922 | .043 | .108 |
| 2 | 8 first-order factors allowed to correlate freely | | 3172 | 2456 | < .001 | .929 | .926 | .042 | .103 |
| 3 | Single-factor model | | 4172 | 2484 | < .001 | .833 | .828 | .064 | .133 |

*Notes.* BRIEF = Behavior Rating Inventory of Executive Function. CFI = Confirmatory Fit Index. TLI = Tucker-Lewis fit Index. RMSEA = Root Mean Square Error of Approximation. SRMR = Standardized Mean Square Residual.

**References**

1. Schermelleh-Engel K, Moosbrugger H, Müller H. Evaluating the fit of structural equation models: Tests of significance and descriptive goodness-of-fit measures. Methods of Psychological Research Online. 2003;8(2):23-74.
